# Biodegradation Resistance and Bioactivity of Hydroxyapatite Enhanced Mg-Zn Composites via Selective Laser Melting

**DOI:** 10.3390/ma10030307

**Published:** 2017-03-17

**Authors:** Cijun Shuai, Yuanzhuo Zhou, Youwen Yang, Pei Feng, Long Liu, Chongxian He, Mingchun Zhao, Sheng Yang, Chengde Gao, Ping Wu

**Affiliations:** 1 State Key Laboratory of High Performance Complex Manufacturing, Central South University, Changsha 410083, China; shuai@csu.edu.cn (C.S.); zyz420357155@csu.edu.cn (Y.Z.); yangyouwen@csu.edu.cn (Y.Y.); fengpei@csu.edu.cn (P.F.); liulong@csu.edu.cn (L.L.); hechongxian@csu.edu.cn (C.H.); 2 Key Laboratory of Organ Injury, Aging and Regenerative Medicine of Hunan Province, Changsha 410008, China; 3 School of Material Science and Engineering, Central South University, Changsha 410083, China; mczhao@csu.edu.cn; 4 Human Reproduction Center, Shenzhen Hospital of Hongkong University, Shenzhen 518053, China; tobyys2000@aliyun.com; 5 College of Chemistry, Xiangtan University, Xiangtan 411105, China

**Keywords:** selective laser melting, heterogeneous nucleation, bone-like apatite, second phase strengthening, biodegradation resistance

## Abstract

Mg-Zn alloys have attracted great attention as implant biomaterials due to their biodegradability and biomechanical compatibility. However, their clinical application was limited due to the too rapid degradation. In the study, hydroxyapatite (HA) was incorporated into Mg-Zn alloy via selective laser melting. Results showed that the degradation rate slowed down due to the decrease of grain size and the formation of protective layer of bone-like apatite. Moreover, the grain size continually decreased with increasing HA content, which was attributed to the heterogeneous nucleation and increased number of nucleation particles in the process of solidification. At the same time, the amount of bone-like apatite increased because HA could provide favorable areas for apatite nucleation. Besides, HA also enhanced the hardness due to the fine grain strengthening and second phase strengthening. However, some pores occurred owing to the agglomerate of HA when its content was excessive, which decreased the biodegradation resistance. These results demonstrated that the Mg-Zn/HA composites were potential implant biomaterials.

## 1. Introduction

Over the past decade, Mg alloys have been known as potential bone implants owing to their biodegradability and suitable elastic modulus [1,2,3]. Among them, Mg-Zn alloys arouse growing interest of researchers because of their excellent mechanical and biological properties [4,5]. Besides, Zn is an essential element in human body and can promote the bone mineralization [6,7]. However, the biomedical application of Mg-Zn alloys is hindered mainly caused by their too rapid degradation, leading to the loss of the mechanical stability before the formation of new bone [8,9].

Hydroxyapatite (HA, Ca_10_(PO_4_)_6_(OH)_2_) possesses a low solubility and can induce the formation of bone-like apatite layer in body environment, which makes it a potential reinforcement for increasing the biodegradation resistance of Mg alloys [10,11,12]. Sunil et al. [13] found that nano hydroxyapatite could increase the corrosion resistance of Mg prepared by friction stir processing (FSP). Campo et al. [14] fabricated the Mg–HA composites via powder metallurgy and discovered that the corrosion resistance of Mg was enhanced by the introduction of HA. They also found some pores resulting from the excessive HA accumulation, which weakened the corrosion resistance.

Selective laser melting (SLM) is a potential method to prepare homogeneous composites due to the rapid melting and solidifying process, which is able to limit the movement of the unmelted phase in the melt pool [15,16,17]. Wei et al. [18] prepared stainless-steel/nano-hydroxyapatite composites by SLM and found that the decrease of solidification time could restrict the HA aggregation. Hao et al. [19] discovered that the porosity of stainless steel/hydroxyapatite composites could be reduced by optimizing the SLM processing parameters. Chi et al. used the SLM technique to fabricate Mg with improved mechanical properties [20,21]. The effect of processing parameters on performance was also investigated [22,23]. Moreover, research into SLM of metals to be used as dental parts and load-bearing implants has been carried out [19,24].

In this study, HA was incorporated into Mg-3Zn alloy via selective laser melting in order to retard its degradation. Then, the effects of HA on the degradation behavior, bioactivity, microstructure and mechanical properties of Mg-3Zn alloy were investigated.

## 2. Results

### 2.1. Microstructure and Composition

**Figure 1:**
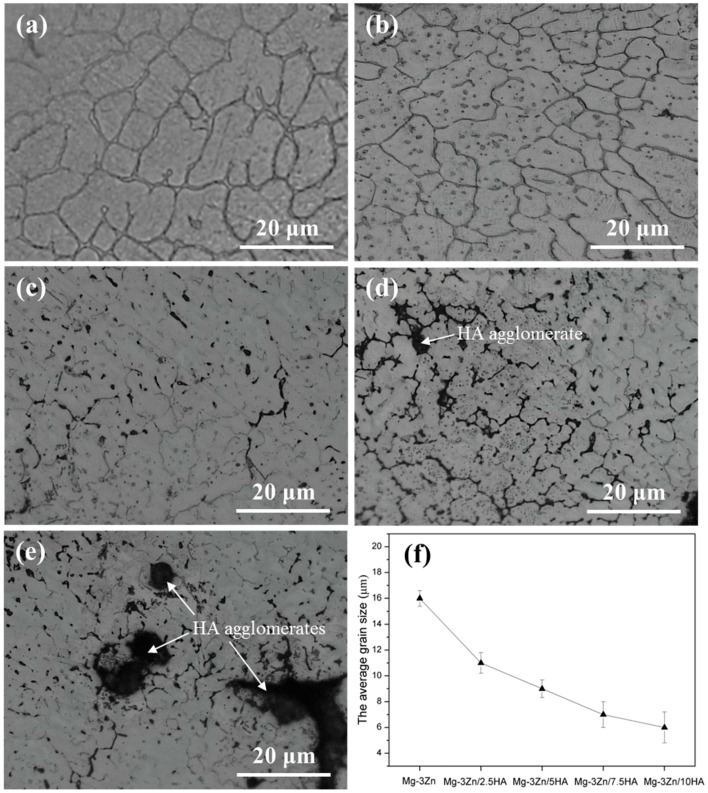
The crystal structure of the Mg-3Zn/*x*HA composites: (**a**) Mg-3Zn alloy; (**b**) Mg-3Zn/2.5HA; (**c**) Mg-3Zn/5HA; (**d**) Mg-3Zn/7.5HA; (**e**) Mg-3Zn/10HA composite and (**f**) their average grain size.

The crystal structure of the Mg-3Zn/*x*HA composites processed by selective laser melting is shown in Figure 1. A relatively large dendrite grain was found in the Mg-3Zn alloy (Figure 1a). A relatively uniform distribution of the HA particles was observed in Mg-3Zn/2.5HA (Figure 1b) and Mg-3Zn/5HA composite (Figure 1c). A few agglomerations of HA particles appeared in the Mg-3Zn/7.5HA composite (Figure 1d), whereas the size and number of the agglomerates significantly increased with further increased HA content (Figure 1e). The porosity of the produced specimens was calculated using the following equation:

% porosity = 100 − % density(1)

The density measurement was based on Archimedes’ law [25]. The porosity of the Mg-3Zn alloy was 2.1%. The porosity of the Mg-3Zn/*x*HA composites with HA content of 2.5, 5, 7.5 and 10 wt. % was 2.3%, 2.7%, 3.3% and 4.5%, respectively. The grain size of the composites was calculated by a linear intercept method [26]. The Mg-3Zn alloy showed a relatively large dendritic structure with an average grain size of 16 μm, while the average grain sizes of the Mg-3Zn/2.5HA, Mg-3Zn/5HA, Mg-3Zn/7.5HA and Mg-3Zn/10HA composites were 11 μm, 9 μm, 7 μm and 6 μm, respectively (Figure 1f). The results showed that the HA particles contributed to the grain refinement of Mg-3Zn alloy, which was attributed to the heterogeneous nucleation in the process of solidification. HA, as a kind of typical ceramic material, had a melting point (1650 °C) [27] much higher than the processing temperature (~700 °C). Also, the crystal of HA and Mg both belonged to a hexagonal system [28]. Thus, the HA particles acted as a heterogeneous nucleus on the liquid/solid interface during solidification, which increased the nucleation rate and significantly decreased the grain size.

**Figure 2:**
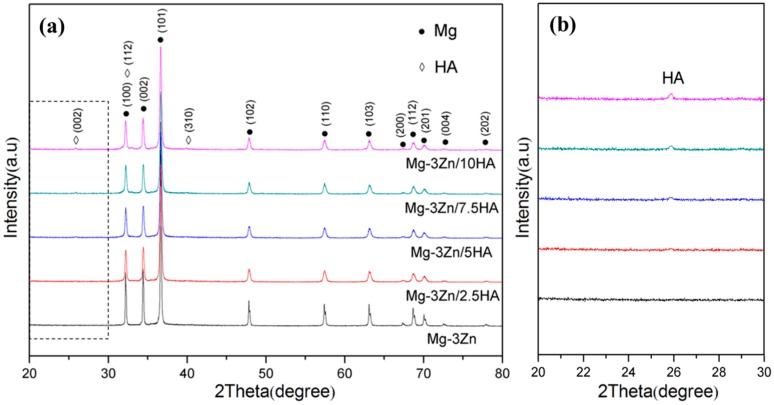
The X-ray diffractometer (XRD) spectra of the Mg-3Zn/*x*HA composites with 2θ range of (**a**) 20°–80° and (**b**) 20–30°.

The X-ray diffractometer (XRD) patterns of the Mg-3Zn/*x*HA composites were shown in Figure 2. There were peaks only corresponding to the α-Mg phase in the XRD pattern of the Mg-3Zn alloy, which might be attributed to the complete solid solution of Zn atoms or the low volume fractions of the precipitation phases. Compared with the XRD pattern of the Mg-3Zn alloy, there were also peaks for HA phase in the XRD pattern of the Mg-3Zn/*x*HA (*x* = 2.5, 5, 7.5 and 10 wt. %) composites (Figure 2a) and the diffraction intensities of HA phase increased with increasing HA content (Figure 2b).

**Figure 3:**
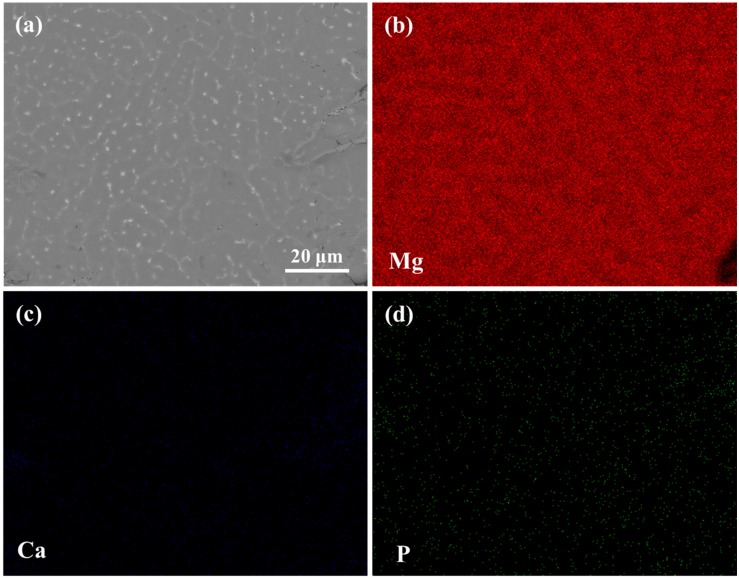
The scanning electron microscopy (SEM) observation and energy dispersive spectroscopy (EDS) analysis of the Mg-3Zn/5HA composite: (**a**) SEM image in back scattered electron mode and corresponding EDS map showing (**b**) Mg; (**c**) Ca and (**d**) P element distribution.

The scanning electron microscopy (SEM) observation combined with energy dispersive spectroscopy (EDS) analysis was conducted to detect the compositional distribution of the Mg-3Zn/5HA composite (Figure 3). HA, mainly composed of Ca and P element, was homogeneously distributed in the composite. During the laser process, the HA particles did not have enough time to accumulate in the melt pools with the rapid solidification rate, which contributed to the homogeneous distribution of HA.

### 2.2. Immersion Tests

**Figure 4:**
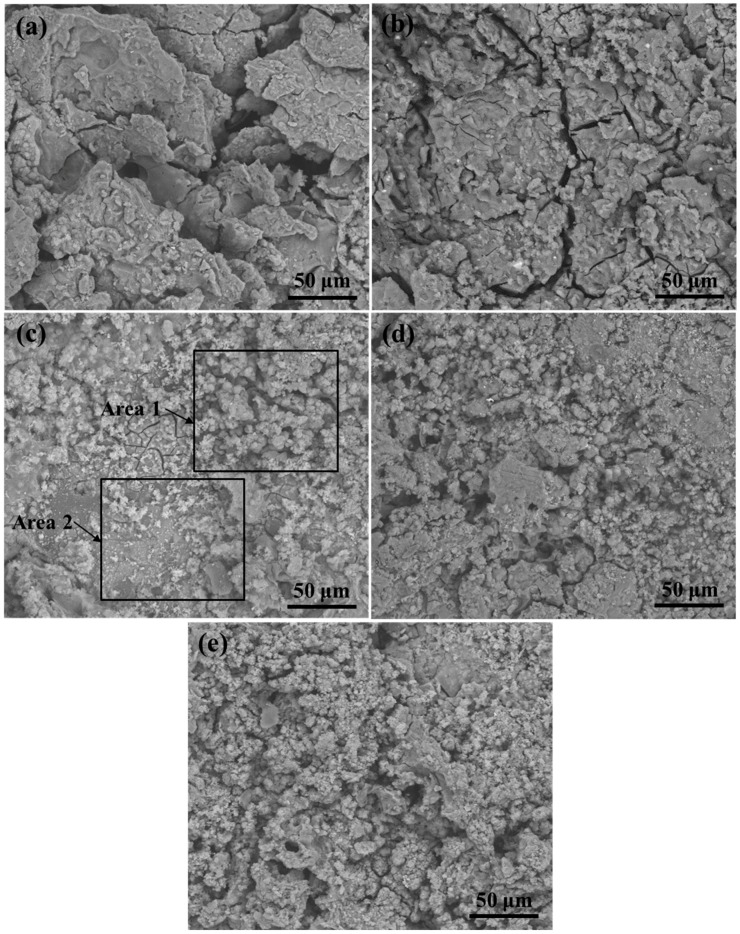
The surface morphology of the Mg-3Zn/*x*HA composites after immersion in the simulated body fluid (SBF) solution for 48 h: (**a**) Mg-3Zn alloy; (**b**) Mg-3Zn/2.5HA; (**c**) Mg-3Zn/5HA; (**d**) Mg-3Zn/7.5HA and (**e**) Mg-3Zn/10HA composite.

The surface morphology of the Mg-3Zn/*x*HA composites immersed in the simulated body fluid (SBF) solution for 48 h was shown in Figure 4. The Mg-3Zn alloy underwent severe local corrosion with obvious deep corrosion pits (Figure 4a). Some precipitates and cracks appeared on the Mg-3Zn/2.5HA composite (Figure 4b). Relatively compact corroded surface and spherical precipitate were observed on the Mg-3Zn/5HA composite (Figure 4c). Besides, the quantity of the spherical precipitate increased with further increased HA content (Figure 4d,e), while some local corrosion occurred at the same time. The appearance of local corrosion on the Mg-3Zn/7.5HA and Mg-3Zn/10HA composites was caused by the defects around the aggregates, through which the SBF solution could penetrate into the composites.

**Figure 5:**
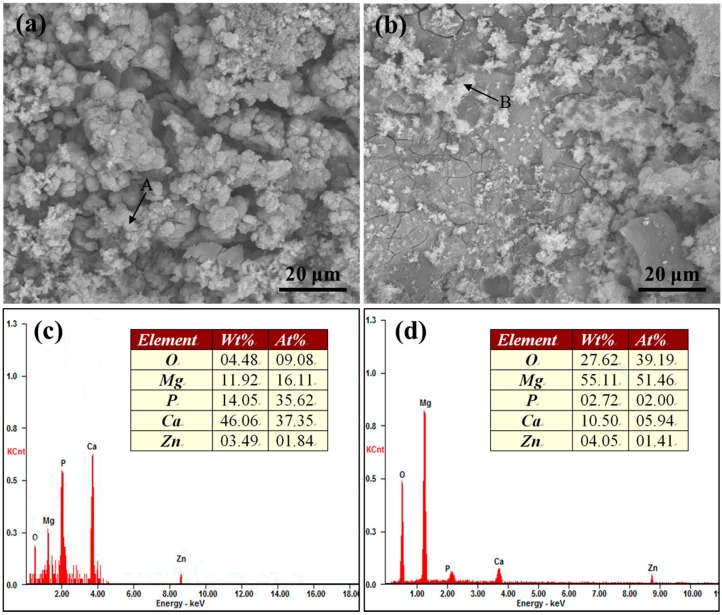
The enlarged view of areas in Figure 4c and EDS result of the precipitates marked by the arrow: enlarged view of area 1 (**a**) and area 2 (**b**); EDS result of the arrow A (**c**) and arrow B (**d**).

The enlarged view of marked areas (Figure 4c) and EDS result of the precipitates are shown in Figure 5. The precipitates in area 1 were spherical (Figure 5a), while the precipitates in area 2 were flocculent (Figure 5b). The EDS results revealed that the spherical precipitates contained 1.84 Zn, 9.08 O, 16.11 Mg, 35.62 P and 37.35 at. % Ca elements (Figure 5c). The flocculent precipitates contained 1.41 Zn, 39.19 O, 51.46 Mg, 2.00 P and 5.94 at. % Ca elements (Figure 5d). Ca and P elements, the main constituent elements of apatite, were rich in the spherical precipitates, which indicated that they were the bone-like apatite. The flocculent precipitates were abundant in Mg and O elements, which implied that they were Mg(OH)_2_. Also, the flocculent precipitates contained some Ca and P elements, which might be derived from the HA of the composite.

**Figure 6:**
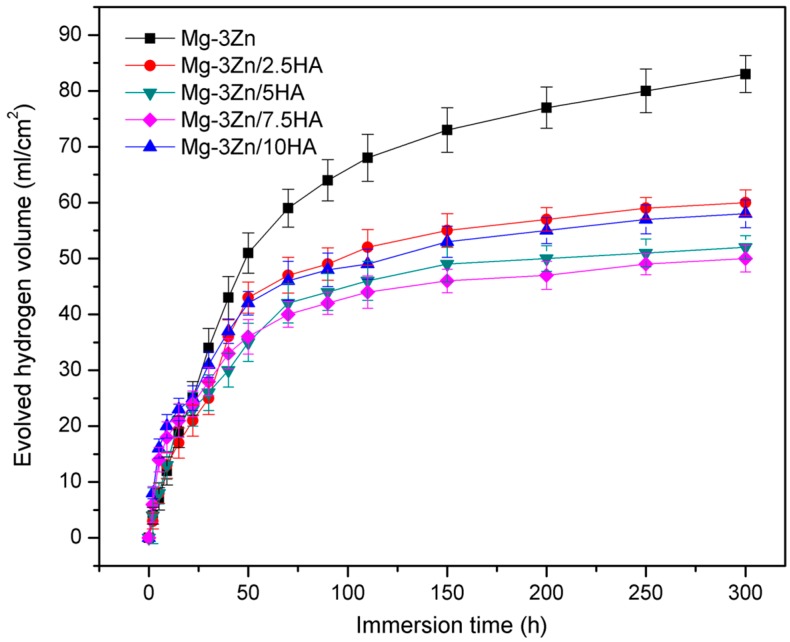
The evolved hydrogen volume of the Mg-3Zn/*x*HA composites immersed in SBF solution at 37 ± 0.5 °C.

The evolved hydrogen volume of the Mg-3Zn/*x*HA composites immersed in SBF solution was measured to investigate the effect of HA on the degradation of the Mg-3Zn alloy (Figure 6). At the early stage of immersion, a larger number of bubbles appeared. With the immersion time increased, corrosion products attached to the surface of the composites and the generated bubbles reduced. After immersion for 300 h, the evolved hydrogen volume of the Mg-3Zn, Mg-3Zn/2.5HA, Mg-3Zn/5HA, Mg-3Zn/7.5HA, Mg-3Zn/10HA composite was 83, 60, 52, 50 and 58 mL/cm^2^, respectively. When the HA content was below 7.5 wt. %, the hydrogen generation was hindered with increasing HA content. This was due to the grain refinement, which promoted the growth of the Mg(OH)_2_ film and thus isolated the substrate from the solution. Moreover, HA could facilitate the formation of stable bone-like apatite layer, which provided a stable protection effect. Unfortunately, with further increasing HA content, the adverse effect of local corrosion caused by the defects around the HA aggregates overshadowed the beneficial effect of the grain refinement and the formation of bone-like apatite, which promoted hydrogen generation.

### 2.3. Mechanical Properties

**Figure 7:**
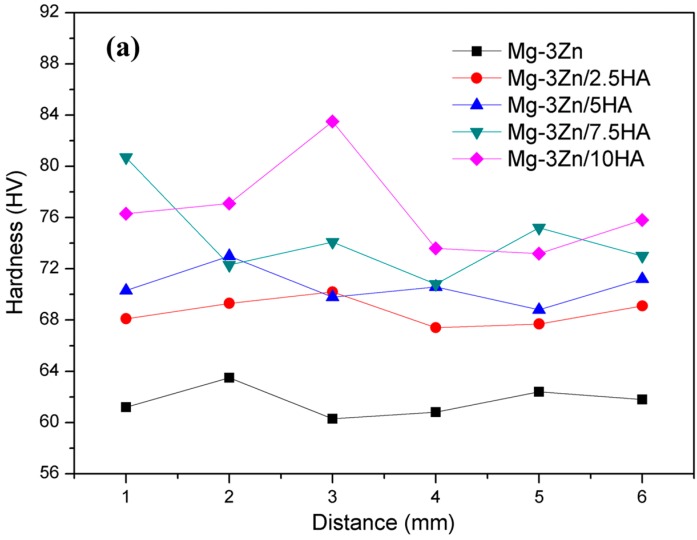

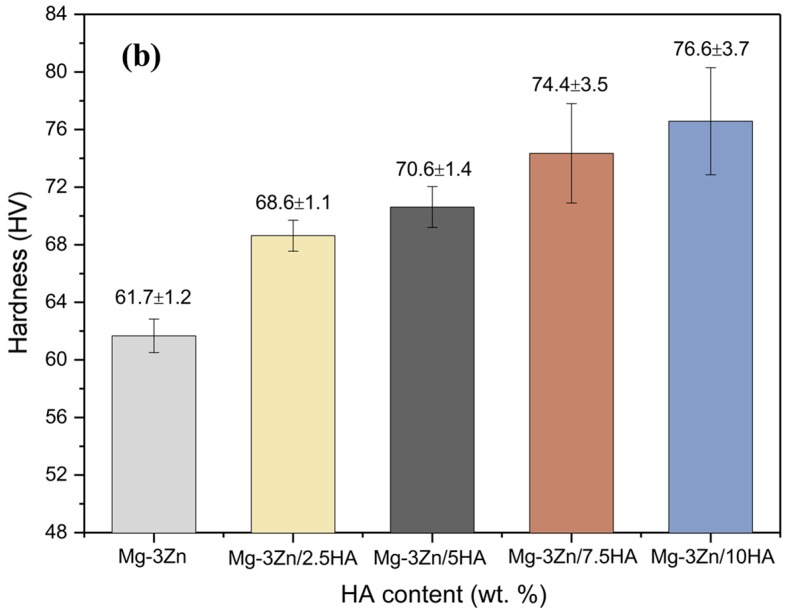
The hardness of the Mg-3Zn/*x*HA composites: (**a**) hardness distribution and (**b**) average hardness.

The hardness of the Mg-3Zn/*x*HA composites is shown in Figure 7. The hardness distribution (Figure 7a) across the cross-section of the Mg-3Zn alloy, Mg-3Zn/2.5HA and Mg-3Zn/5HA composites showed a slight variance. The average hardness of the laser-melted Mg-3Zn alloy was 62 HV, which was higher than that of the as-extruded Mg-3Zn alloy (55 HV) [29]. However, a significant variation in the hardness distribution was observed in the Mg-3Zn/7.5HA and Mg-3Zn/10HA composites, which might be caused by the large agglomerate of HA. Besides, it could be found that the hardness of Mg-3Zn/*x*HA composites was higher than that of Mg-3Zn alloy and their hardness increased with increasing HA content (Figure 7b). The grain refinement had certainly contributed to the enhancement of hardness, which could be explained by the Hall–Petch equation. Besides, the HA particles existed at the grain boundary of the composites were stiff, so that the movement of the dislocations had to bypass the HA particles. Thus, the hardness of matrix was enhanced with the increase in HA content.

## 3. Discussion

**Figure 8:**
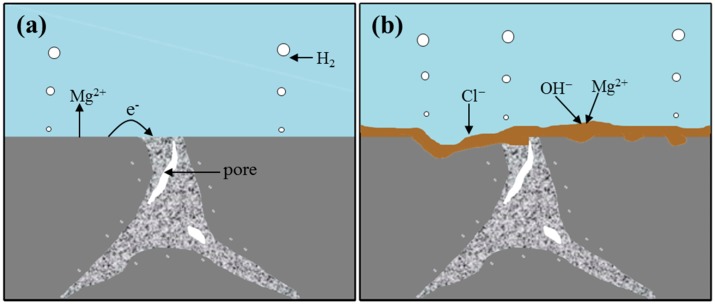

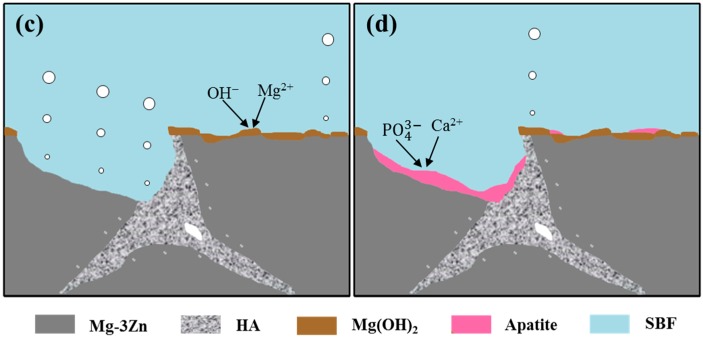
The schematic diagram displaying the degradation mechanism of the Mg-3Zn/*x*HA composites.

At the beginning of the immersion, the SBF solution was in direct contact with the Mg-3Zn/*x*HA composite. The reaction between the solution and composite was relatively intense in this stage. The products were mainly Mg^2+^ and H_2_ (Figure 8a). As the extension of immersion time, the generated Mg^2+^ combined with OH^−^ in the solution to form Mg(OH)_2_ films, which could protect the composite in a certain extent (Figure 8b). However, the Mg(OH)_2_ protective films could react with Cl^−^ in the solution and then transformed into a soluble MgCl_2_, which undermined the protective effect. As the immersion time further increased, the solution penetrated into the composite, which leaded to partial shedding of substrate (Figure 8c). Then, HA in the composite was exposed to the solution, which offered favorable areas for apatite nucleation (Figure 8d). This could be explained by the Kim’s theory [30]. HA possessed negative surface charge due to the existence of hydroxyl and phosphate groups. While in the SBF solution, OH^−^ and ${\mathrm{HPO}}_{4}^{2-}$ ions combined with Ca^2+^ ions to form Ca-rich amorphous calcium phosphate (ACP) on HA. The Ca-rich ACP, which possessed positive surface charge, could further interacted with the negative phosphate ion in the solution to form Ca-poor ACP, which finally crystallized into the bone-like apatite.

## 4. Materials and Methods

### 4.1. Materials

**Figure 9:**
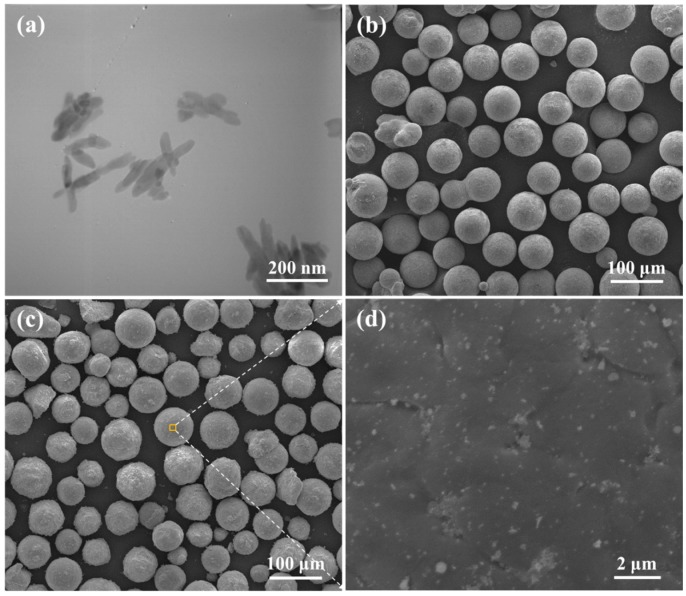
The raw and mixed powder: (**a**) transmission electron microscopy (TEM) image of the raw HA powder; (**b**) SEM image of the raw Mg-3Zn powder; (**c**) SEM image of the Mg-3Zn/5HA mixed powder and (**d**) an enlarged view of marked area in Figure 9c.

The HA powder (Figure 9a) was purchased from the Nanjing Emperor Nano Material Co., Ltd. (Nanjing, China) with a needle-like shape (a width of 20 nm and a length of 150 nm). The Mg-3Zn powder (Figure 9b) was obtained from the Hao Tian Nano Science and Technology Co., Ltd. (Shanghai, China) with the average particle size of approximately 60 μm and nominal chemical composition of 3.16 wt. % Zn, 0.48 wt. % Zr and balanced Mg. The particle morphologies of the HA and Mg-3Zn raw powders were investigated using transmission electron microscopy (TEM, JEM2100, JEOL, Beijing, China) and scanning electron microscopy (SEM, MIRA3 LMU, TESCAN, Brno, Czech Republic), respectively. The Mg-3Zn/*x*HA (0, 2.5, 5, 7.5 and 10 wt. % of HA, remaining being Mg-3Zn) powders were respectively milled for 4 h in a ball mill (DECO-PBM-V-0.4L, DECODK CO., Ltd., Changsha, China) under the protection of SF_6_ and CO_2_ gas. The mixed powders keep the spherical shape, with the HA particles distributed on the surface of Mg-3Zn powder (Figure 9c,d).

### 4.2. Processing

The home-made laser melting system was used to prepare the Mg-3Zn/*x*HA composites [31]. The system included a fiber laser (maximum output power was 110 W), an optical focusing system, a motion platform and a gas shielding device. The processing parameters in this work were: the laser power was 65 W, the scanning speed was 10 mm/s and the laser spot diameter was 0.2 mm. The samples were produced in multi-tracks and multi-layers under the protection of argon gas. The overlapping rate was 20% and the thickness of each layer was 0.1 mm. The Hatch spacing was 0.16 mm. The specimen dimensions were 10 × 10 × 5 mm^3^. And the samples were observed in the section parallel to deposition direction.

### 4.3. Microstructure

The Mg-3Zn/*x*HA composites were ground with abrasive papers, polished with diamond grits, and then ultrasonically cleaned in absolute ethanol, respectively. The optical microscopy (ICC50W, LEICA, Brunswick, Germany) was employed to observe their crystal structure. The X-ray diffractometer (XRD, AXS D8, Bruker AXS Inc., Bruker, Germany) was applied to characterize their phase composition. The XRD spectrum was recorded at a scanning rate of 10°/min with 2θ range of 20° to 80°. Besides, a slow scanning rate of 1°/min was applied with 2θ range of 20° to 30° to observe the change of HA diffraction peak. The SEM coupled with energy dispersive spectroscopy (EDS) was employed to detect the element distribution.

### 4.4. Immersion Tests

Immersion experiments were performed in SBF solution [32] with an ion concentration similar to human plasma at 37 ± 0.5 °C to evaluate the degradation behavior of the Mg-3Zn/*x*HA composites. The released hydrogen volume was measured during the immersion. The dimensions of the composites were 10 × 10 × 5 mm^3^.

The surface morphology and the precipitation on the composites after immersion in SBF solution for 48h were characterized by SEM coupled with EDS.

### 4.5. Mechanical Properties

The Vickers hardness of the Mg-3Zn/*x*HA composites was measured by the HMV-2T Vickers hardness tester (Shanghai Zhuoji Instruments Co. Ltd., Shanghai, China) with a load of 9.8 N and a loading time of 15 s. Each sample was measured from the center to the edge to get six measurements, and its average was considered as the Vickers hardness.

### 4.6. Statistical Analysis

The data were shown as mean ± standard deviation (SD) and were obtained from six independent specimens. Statistical analysis was applied to estimate the data difference by the SPSS software. Difference was considered significant when *p* < 0.05.

## 5. Conclusions

The Mg-3Zn/HA composites were developed to be used as potential implant biomaterials. The HA was introduced into Mg-3Zn alloy by laser rapid melting. The grain size of Mg-3Zn/HA composites decreased with increasing HA content, owing to the heterogeneous nucleation and increased number of nucleation particles in the process of solidification. The biodegradation resistance of the composites was increased at the same time, which was attributed to the decrease of grain size and the formation of stable apatite protective layer. Additionally, the hardness of the Mg-3Zn alloy was enhanced due to the fine grain strengthening and second phase strengthening.

## References

[1] Chen Y., Xu Z., Smith C., Sankar J. (2014). Recent advances on the development of magnesium alloys for biodegradable implants. Acta Biomater..

[2] Xin Y., Hu T., Chu P. (2011). In vitro studies of biomedical magnesium alloys in a simulated physiological environment: A review. Acta Biomater..

[3] Rad H., Idris M., Kadir M., Farahany S. (2011). Microstructure analysis and corrosion behavior of biodegradable Mg–Ca implant alloys. Mater. Des..

[4] Li H., Peng Q., Li X., Li K., Han Z., Fang D. (2014). Microstructures, mechanical and cytocompatibility of degradable Mg-Zn based orthopedic biomaterials. Mater. Des..

[5] Cai S., Lei T., Li N., Feng F. (2012). Effects of Zn on microstructure, mechanical properties and corrosion behavior of Mg-Zn alloys. Mater. Sci. Eng. A Struct..

[6] Li J., Cao P., Zhang X., Zhang S., He Y. (2010). In vitro degradation and cell attachment of a PLGA coated biodegradable Mg–6Zn based alloy. J. Mater. Sci..

[7] Luo X., Barbieri D., Davison N., Yan Y., Bruijn J., Yuan H. (2014). Zinc in calcium phosphate mediates bone induction: In vitro and in vivo model. Acta Biomater..

[8] González S., Pellicer E., Fornell J., Blanquer A., Barrios L., Ibáñez E., Solsona P., Suriñach S., Baró M., Nogués C. (2012). Improved mechanical performance and delayed corrosion phenomena in biodegradable Mg-Zn-Ca alloys through Pd-alloying. J. Mech. Behav. Biomed..

[9] Yu K., Chen L., Zhao J., Li S., Dai Y., Huang Q., Yu Z. (2012). In vitro corrosion behavior and in vivo biodegradation of biomedical β-Ca_3_(PO_4_)_2_/Mg-Zn composites. Acta Biomater..

[10] Zhang J., Dai C., Wei J., Wen Z. (2012). Study on the bonding strength between calcium phosphate/chitosan composite coatings and a Mg alloy substrate. Appl. Surf. Sci..

[11] Chen X., Birbilis N., Abbott T. (2011). A simple route towards a hydroxyapatite–Mg(OH) conversion coating for magnesium. Corros. Sci..

[12] Mróz W., Bombalska A., Burdyńska S., Jedyński M., Prokopiuk A., Budner B., Ślósarczyk A., Zima A., Menaszek E., Ścisłowska-Czarnecka A. (2010). Structural studies of magnesium doped hydroxyapatite coatings after osteoblast culture. J. Mol. Struct..

[13] Ratna Sunil B., Sampath Kumar T., Chakkingal U., Nandakumar V., Doble M. (2014). Friction stir processing of magnesium-nanohydroxyapatite composites with controlled in vitro degradation behavior. Mat. Sci. Eng. C Mater..

[14] Campo R., Savoini B., Muñoz A., Monge M., Garcés G. (2014). Mechanical properties and corrosion behavior of Mg–HAP composites. J. Mech. Behav. Biomed..

[15] Contuzzi N., Campanelli S., Ludovico A. (2011). 3D Finite Element Analysis in the selective laser melting process. Int. J. Simul. Mod..

[16] Li R., Shi Y., Liu J., Xie Z., Wang Z. (2010). Selective laser melting W–10 wt.% Cu composite powders. Int. J. Adv. Manuf. Tech..

[17] Mróz W., Budner B., Syroka R., Niedzielski K., Golański G., Slósarczyk A., Schwarze D., Douglas T. (2015). In vivo implantation of porous titanium alloy implants coated with magnesium-doped octacalcium phosphate and hydroxyapatite thin films using pulsed laser depostion. J. Biomed. Mater. Res. B.

[18] Wei Q., Li S., Han C., Li W., Cheng L., Hao L., Shi Y. (2015). Selective laser melting of stainless-steel/nano-hydroxyapatite composites for medical applications: Microstructure, element distribution, crack and mechanical properties. J. Mater. Process. Technol..

[19] Hao L., Dadbakhsh S., Seaman O., Felstead M. (2009). Selective laser melting of a stainless steel and hydroxyapatite composite for load-bearing implant development. J. Mater. Process. Technol..

[20] Ng C., Savalani M., Man H. (2011). Fabrication of magnesium using selective laser melting technique. Rapid Prototyp. J..

[21] Ng C., Savalani M., Lau M., Man H. (2011). Microstructure and mechanical properties of selective laser melted magnesium. Appl. Surf. Sci..

[22] Wei K., Gao M., Wang Z., Zeng X. (2014). Effect of energy input on formability, microstructure and mechanical properties of selective laser melted AZ91D magnesium alloy. Mater. Sci. Eng. A.

[23] Pawlak A., Rosienkiewicz M., Chlebus E. (2017). Design of experiments approach in AZ31 powder selective laser melting process optimization. Arch. Civ. Mech. Eng..

[24] Vandenbroucke B., Kruth J. (2007). Selective laser melting of biocompatible metals for rapid manufacturing of medical parts. Rapid Prototyp. J..

[25] Schubert T., Trindade B., Weißgärber T., Kieback B. (2008). Interfacial design of Cu-based composites prepared by powder metallurgy for heat sink applications. Mater. Sci. Eng. A.

[26] Dini G., Najafizadeh A., Ueji R., Monir-Vaghefi S. (2010). Tensile deformation behavior of high manganese austenitic steel: The role of grain size. Mater. Des..

[27] Fadli A., Sopyan I., Singh R. (2012). Porous Alumina from Protein Foaming-Consolidation Method Containing Hydrothermal Derived Hydroxyapatite Powder. Appl. Mech. Mater..

[28] Laurencin D., Almora-Barrios N., Leeuw N., Gervais C., Bonhomme C., Mauri F., Chrzanowski W., Knowles J., Newport R., Wong A. (2011). Magnesium incorporation into hydroxyapatite. Biomaterial.

[29] Maeng D., Kim T., Lee J., Hong S., Seo S., Chun B. (2000). Microstructure and strength of rapidly solidified and extruded Mg-Zn alloys. Scripta Mater..

[30] Kim H., Himeno T., Kokubo T., Nakamura T. (2005). Process and kinetics of bonelike apatite formation on sintered hydroxyapatite in a simulated body fluid. Biomaterial.

[31] Yang Y., Wu P., Lin X., Liu Y., Bian H., Zhou Y., Gao C., Shuai C. (2016). System development, formability quality and microstructure evolution of selective laser-melted magnesium. Virtual Phys. Prototyp..

[32] Hu J., Wang C., Ren W., Zhang S., Liu F. (2010). Microstructure evolution and corrosion mechanism of dicalcium phosphate dihydrate coating on magnesium alloy in simulated body fluid. Mater. Chem. Phys..

